# Public Perceptions and Attitudes Toward COVID-19 Nonpharmaceutical Interventions Across Six Countries: A Topic Modeling Analysis of Twitter Data

**DOI:** 10.2196/21419

**Published:** 2020-09-03

**Authors:** Caitlin Doogan, Wray Buntine, Henry Linger, Samantha Brunt

**Affiliations:** 1 Department of Human Centred Computing Faculty of Information Technology Monash University Caulfield Australia; 2 Department of Data Science and AI Faculty of Information Technology Monash University Clayton Australia; 3 Royal Perth Hospital Perth Australia

**Keywords:** COVID-19, SARS-CoV-2, topic modeling, nonpharmaceutical interventions, social media, public health, machine learning, social distancing, lockdown, face masks, infodemiology

## Abstract

**Background:**

Nonpharmaceutical interventions (NPIs) (such as wearing masks and social distancing) have been implemented by governments around the world to slow the spread of COVID-19. To promote public adherence to these regimes, governments need to understand the public perceptions and attitudes toward NPI regimes and the factors that influence them. Twitter data offer a means to capture these insights.

**Objective:**

The objective of this study is to identify tweets about COVID-19 NPIs in six countries and compare the trends in public perceptions and attitudes toward NPIs across these countries. The aim is to identify factors that influenced public perceptions and attitudes about NPI regimes during the early phases of the COVID-19 pandemic.

**Methods:**

We analyzed 777,869 English language tweets about COVID-19 NPIs in six countries (Australia, Canada, New Zealand, Ireland, the United Kingdom, and the United States). The relationship between tweet frequencies and case numbers was assessed using a Pearson correlation analysis. Topic modeling was used to isolate tweets about NPIs. A comparative analysis of NPIs between countries was conducted.

**Results:**

The proportion of NPI-related topics, relative to all topics, varied between countries. The New Zealand data set displayed the greatest attention to NPIs, and the US data set showed the lowest. The relationship between tweet frequencies and case numbers was statistically significant only for Australia (*r*=0.837, *P*<.001) and New Zealand (*r*=0.747, *P*<.001). Topic modeling produced 131 topics related to one of 22 NPIs, grouped into seven NPI categories: Personal Protection (n=15), Social Distancing (n=9), Testing and Tracing (n=10), Gathering Restrictions (n=18), Lockdown (n=42), Travel Restrictions (n=14), and Workplace Closures (n=23). While less restrictive NPIs gained widespread support, more restrictive NPIs were perceived differently across countries. Four characteristics of these regimes were seen to influence public adherence to NPIs: timeliness of implementation, NPI campaign strategies, inconsistent information, and enforcement strategies.

**Conclusions:**

Twitter offers a means to obtain timely feedback about the public response to COVID-19 NPI regimes. Insights gained from this analysis can support government decision making, implementation, and communication strategies about NPI regimes, as well as encourage further discussion about the management of NPI programs for global health events, such as the COVID-19 pandemic.

## Introduction

SARS-CoV-2, the novel virus causing COVID-19, was declared a pandemic by the World Health Organization (WHO) on March 11, 2020. SARS-CoV-2 has infected millions of people worldwide [1] and continues to threaten population health, as well as the socioeconomic and geopolitical positions, of many countries. In the absence of preventative and curative pharmaceutical treatments specific to COVID-19, governments are reliant on the success of strategic response programs to mitigate, delay, or suppress the transmission of SARS-CoV-2 [2]. Typically, these programs are dynamic regimes of nonpharmaceutical interventions (NPIs) [3]. NPIs are public health measures, aside from the use of pharmaceuticals or vaccinations, deployed by governments and health authorities to control community transmission of disease [3,4].

The restrictiveness of COVID-19 NPI regimes is dependent on a government's strategy to control the transmission of SARS-CoV-2 (eg, elimination versus suppression) [5]. However, for these strategies to be effective, the public must maintain adherence to the prescribed NPIs. Governments and health authorities need to ensure the ongoing public adherence to NPIs to gain control of viral transmission. To do so requires a greater understanding of the trends in the public’s perceptions and attitudes toward these NPI regimes, as well as a means of determining why, and in what contexts, these adherence behaviors arise, decrease, and persist [6]. This level of understanding could assist governments in making more informed decisions about NPIs so that they may be made more acceptable to the public. Studies have demonstrated that NPI regimes with a higher degree of public acceptability attract a greater level of public adherence and, ultimately, reduce the rates of infection within a community [7,8].

Understanding public attitudes toward NPIs, together with epidemiological data, may also inform the optimal time when restrictions could be eased or removed, and importantly, how these adjustments are communicated to the public [9]. This knowledge will be imperative in the event these strategies may need to be implemented again to suppress any subsequent waves of infection during later phases of the COVID-19 pandemic.

Epidemiological modeling [10], surveys [2], and the analysis of past pandemics [11] have been the primary means to support decision making around COVID-19 NPI regimes. Several studies have assessed the success of such programs in countries that have passed the peak of infections [12,13], offering valuable lessons for other countries battling the virus [12]. However, a more comprehensive understanding of the public's perceptions and attitudes about NPIs would be of significant additional benefit. Moreover, the need for expedient implementation of NPIs requires rapid analysis. Social media analysis offers this opportunity due to the widespread use of these platforms, and the relative speed of data capture [14].

Recent studies have demonstrated that social media mining could provide information about the public response to specific health measures, such as suspension of sporting matches [15], cancellation of festivals [16], and provisions for vulnerable groups [17]. Our study can further contribute to this literature by demonstrating that social media mining can derive rapid insights about public perceptions and attitudes to NPI regimes.

Given the need for both timely responses to outbreaks [15] and more in-depth insights about trends in the public perception of NPIs, we chose to collect Twitter data, as it is accessible and of sufficient volume for the computational and qualitative analysis needed to provide information on attitudinal trends. Moreover, a comparative analysis of data drawn from multiple geographic regions may assist in the identification of factors that contribute to the understanding of the public acceptability and, ultimately, sustained adherence to NPI regimes.

In this study, we used a hybrid computational approach to analyze English language tweets related to COVID-19 NPIs across six anglophone countries. The aims of this study are to (1) identify which NPIs attract public attention in each of the countries and the extent to which they do so, (2) describe the perceptions and attitudes toward these NPIs and compare these between countries, (3) identify factors that may influence public perception and attitudes to NPI regimes.

## Methods

This study adopts a hybrid approach that integrates computational and qualitative techniques to describe the public’s perceptions and attitudes toward NPIs.

### Data Collection and Preprocessing

Using the Twitter streaming API (application programming interface) service, we collected 2,587,625 tweets posted between January 1 and April 30, 2020. During the 121 days surveyed, COVID-19 was declared a pandemic, and each of the chosen countries had implemented NPI regimes to control the spread of the virus. We considered varying the collection start times for each country but felt there was no strong justification for doing so. The major onset of significant cases was within a week for all the chosen countries and, given that media coverage was international, the onset of tweeting predated the onset of cases in all countries. Tweets were retrieved using three hashtags related to COVID-19 in each country to collect an initial pool of data. We did not include hashtags such as #COVID-19, as this would not allow for an analysis targeted to one particular country. A frequency analysis of cocurrent hashtags informed the choice of secondary hashtags used for further retrieval of tweets. These hashtags, and the results of the frequency analysis, are reported in Multimedia Appendix 1.

Preprocessing of tweets included duplicate removal, lower casing, contraction expansion, and elongation reduction. Nonalphabetic characters, @usernames, #hashtags, URLs, the queried hashtag, and the name of the specific country were removed. The Python package NLTK [18] was used for tokenization, part-of-speech tagging, lemmatization, and stop-word removal. Using the langid package [19], we identified and removed non-English tweets. Bigrams and trigrams were reviewed for inclusion. Tokens appearing less than 10 times in the data set or tweets containing less than 10 tokens were excluded from further analysis. In total, 777,869 tweets met the criteria for analysis.

### Analysis of Public Attention

In the first instance, we determined if the attention of the public, as given by the number of daily tweets, was related to the number of daily confirmed cases. For each data set, we conducted a Pearson correlation analysis to determine if there was a statistically significant relationship between these variables. Graphical representations of these relationships are presented in Multimedia Appendix 2, and the results of this correlation analysis are presented in Multimedia Appendix 3.

### Topic Modeling and Evaluation

Topic modeling [20] is used to determine hidden (latent) semantic structures that link documents through the identification of commonly co-occurring word sets. Ranked word sets, or topics, are summative representations of the documents in which they appear. We used MetaLDA [21], a topic model empirically demonstrated to perform better than the popular model Latent Dirichlet Allocation (LDA) [22] when modeling tweets that are sparse and noisy texts [23]. The choice of how many topics (k) to construct is a parameter of MetaLDA and affects the quality of the models produced. A quality model is one with coherent and interpretable topics. However, as the best k is not known, multiple models with different values for k (k = {30,40 … 130}) were constructed for each data set [24]. The values of k and the number of models constructed was determined by the size of the data set. The selection of quality models is informed by statistical and qualitative evaluation.

Statistical evaluation of models was based on the coherence measure normalized pointwise mutual information (NPMI) [25], calculated using the Palmetto package [26], and Glove2Vec embeddings [27]. Embeddings were trained on the Wikipedia corpus for all data sets except the Australian and New Zealand data sets, which were trained on a 150-million-word corpus of Australian news articles. We report these evaluations in Multimedia Appendix 4. Models with higher mean NPMI scores were selected for a qualitative review of their topics. The models with the most coherent and interpretable topics were further analyzed.

### Comparative Analysis

Qualitative analysis of topics involved the construction of coding schema to guide the identification and labeling of NPI-related topics. This schema is displayed in Multimedia Appendix 5. The schema was primarily informed by the WHO advisory framework for influenza pandemic NPIs [11], supported by academic literature, and the specific COVID-19 NPI regimes being employed by the six countries at the time of analysis. In this process, we reviewed up to 100 of the most representative tweets per topic to determine if the topic was about NPIs and, if so, labeled with the relevant NPI. We also examined topics for inclusion when these topics referenced NPIs in related topics. Within the research team, we cross-checked the NPI labels assigned to topics [28]. Multimedia Appendix 4 displays the number of topics associated with NPIs for each country.

A comparative analysis of the public discussion of NPIs between countries was conducted. Tweets were recontextualized by reviewing embedded hypermedia, replies, and linked media. We supplemented our analysis by surveying COVID-19–related information provided by each country’s government during the study period. Heatmaps were constructed to identify which NPIs attracted the public’s attention for each country and the extent to which they did so. The proportion of each country’s discussion of an NPI was calculated for each data set as the total number of tweets assigned to topics about an NPI, relative to the total number of tweets assigned to all topics about NPIs. To explore further, we constructed chord diagrams to visualize the relationships between discussions of NPIs in each country. These diagrams, shown in Multimedia Appendix 2, illustrate the number of tweets shared between categories of NPIs.

## Results

### Analysis of Public Attention

A Pearson correlation analysis showed that the number of daily COVID-19 cases and the daily number of tweets were strongly correlated only in the Australian (*r*=0.837, *P*<.001) and New Zealand (*r*=0.747, *P*<.001) data sets for the 121 days surveyed. The analysis was also conducted based on the date of the first confirmed case in each country. Again, a strong correlation was seen only for Australia (*r*=0.823, *P*<.001) and New Zealand (*r*=0.666, *P*<.001). The results of this analysis are shown in Multimedia Appendix 3.

Graphically, it appears that public attention dissipated over time, despite the number of cases continuing to rise exponentially in the surveyed countries except for Australia and New Zealand. These two countries brought their relatively small number of cases under control during the study period, which may account for the correlation between the decline in tweets and cases. Scaled visualizations of the number of tweets and cases per day for each data set are displayed in Multimedia Appendix 2.

### Comparative Analysis

In total, 131 NPI-related topics discussed one of 22 NPIs that were identified. These NPIs were categorized into seven types of NPIs: Personal Protection (n=15), Social Distancing (n=9), Testing and Tracing (n=10), Gathering Restrictions (n=18), Lockdown (n=42), Travel Restrictions (n=14), and Workplace Closures (n=23). The qualitative details of these topics are in Multimedia Appendix 4.

The following discussion details the results of a comparative analysis of the public’s perceptions and attitudes of NPIs between countries. We have divided the analysis and accompanying graphical representations into less restrictive and more restrictive NPIs based on their level of intrusiveness and economic cost. Exemplar tweets that illustrate public perceptions of NPIs are in Multimedia Appendix 6.

#### Analysis of Less Restrictive NPIs

**Figure 1:**
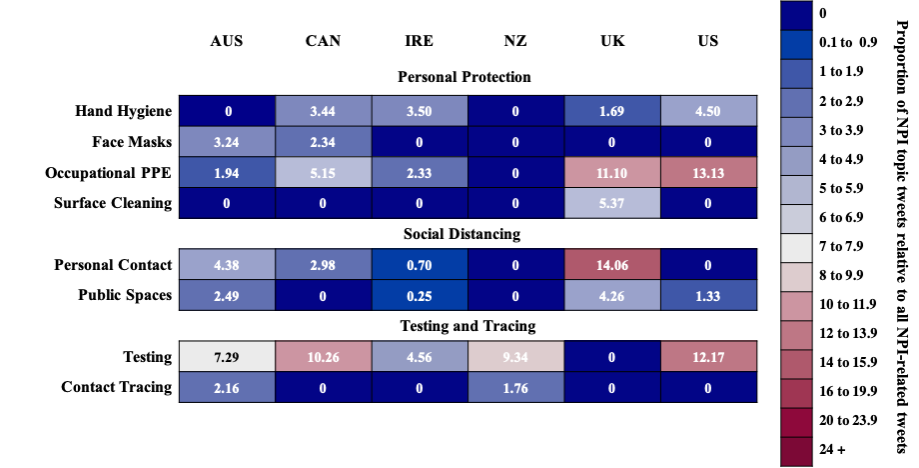
A heatmap of the proportions of tweets associated with less restrictive nonpharmaceutical interventions (NPIs) for each country. PPE: personal protective equipment.

Less restrictive NPIs include Personal Protection, Social Distancing, and Testing and Tracing. The proportions of tweets associated with these three categories are shown in Figure 1.

### Personal Protection

Four NPIs about Personal Protection were identified: Hand Hygiene, Face Masks, Occupational Personal Protective Equipment (PPE), and Surface Cleaning.

Tweets about hand hygiene were concentrated in the early period of each country’s response. While the proportion of topics was relatively low, hand hygiene often featured in tweets discussing more prominent NPIs. Tweets from the United Kingdom referenced the National Health Service’s (NHS) recommendation that hand washing length should equate with the amount of time it takes to sing the “Happy Birthday” song [29]. Tweets in the United States tended to be informative, explaining that soap and warm water was better than hand sanitizers to kill SARS-CoV-2 [30] and that people should stop touching their faces. In contrast, UK tweets expressed frustration at low handwashing compliance and public preference for alcohol-based hand sanitizers. Similarly, Canadian tweets promoted handwashing over the use of disposable gloves and considered those misusing gloves to be irresponsible.

Despite the Australian government’s advice that face masks were not generally necessary for the public [31], Australian tweets perceived face masks to be an important defense against transmission. Canadian tweets expressed confusion over changing government advice regarding the wearing of face masks.

Except for tweets from New Zealand, the public perceived the shortage of occupational PPE to be the result of poor government decision making. Tweets from the United States expressed alarm at these shortages and frequently referenced ongoing media coverage of PPE shortages in outbreak areas. Perceptions diverged, however, as the supply of Chinese-sourced PPE increased in different countries. Irish tweets celebrated the highly publicized shipments, while Australian tweets expressed skepticism of the political motivations behind these deliveries. Canadian tweets voiced concern at the quality of Chinese PPE and lamented the lack of national capacity for PPE manufacturing.

In response to a shortage of cleaning products [32], Canadian and UK tweets encouraged surface cleaning, shared recipes for homemade disinfectants, and discussed practical measures to reduce surface transmission, including cleaning mobile phones.

### Social Distancing

Two NPIs about Social Distancing were identified: Personal Contact and Public Spaces.

UK and Irish tweets discouraged handshaking and reported the rapid adoption of noncontact greetings, including elbow-bumping and foot tapping. However, UK tweets found the cessation of handshaking redundant in full-contact sports given the high degree of bodily contact. The UK discussion of personal contact was highly concentrated, with most tweets reminding others to be mindful of keeping their distance, particularly from the elderly.

Transmission in public spaces, such as on public transport, was perceived as a threat by the Australian, Irish, UK, and US public. US and UK tweets expressed dissatisfaction with the solutions authorities had imposed, such as reducing carriage capacity in London's underground rail services. Irish tweets complained about the challenges of social distancing, given the narrow footpaths in cities. Australian tweets supported social distancing but questioned the arbitrary nature of a 1.5 m (5 ft) specification rather than 2 m (6.5 ft) [33] seen in other countries. Australian and Irish tweets questioned the logic of social distancing when other NPI implementations did not account for the practice, particularly in specific workplaces.

### Testing and Tracing

Two NPIs about Testing and Tracing were identified: Testing and Tracing Apps.

The highest proportions of topics about testing were from the Canadian and US data sets, where the public perception of testing efforts was negative. This was due to inconsistent testing criteria, testing backlogs, faulty testing kits, costs, and a lack of access to tests. Australian tweets viewed the reports of low community transmission to be a statistical manipulation, given testing at that time was restricted to returning travelers. Similarly, New Zealand and Irish tweets questioned the validity of case reports given the testing criteria.

The Australian and New Zealand public expressed concern about the privacy implications of contact tracing apps. The public understood the specifics of data collection, but tweets expressed distrust of tech companies that would be engaged to store the data. New Zealand tweets considered the implications of centralized data models and called for open source code and an independent privacy assessment.

#### Analysis of Restrictive NPIs

**Figure 2:**
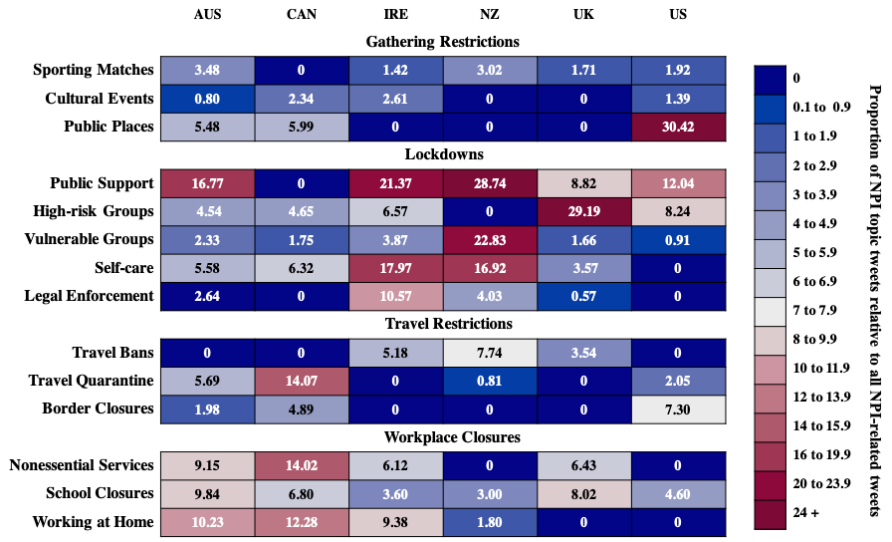
A heatmap of the proportion of tweets associated with restrictive nonpharmaceutical interventions (NPIs) for each country.

Categories of restrictive NPIs include Gathering Restrictions, Lockdowns, Travel Restrictions, and Workplace Closures. The proportions of tweets associated with these three categories are shown in Figure 2.

### Gathering Restrictions

Three NPIs about Gathering Restrictions were identified: Sporting Matches, Cultural Events, and Public Places.

US tweets had a mixed response to the suspension of several major US sporting leagues announced on March 12, 2020. Though disappointed, many accepted the measure as necessary, while others perceived it to be an overreaction. The latter response could be explained by the low number of cases in several states at that time. Except for New Zealand, where the public looked forward to the resumption of elite sports, the suspension of sporting seasons was discussed with similarly disappointed acceptance. UK tweets expressed shock at the sudden cancellation of the English Premier League season. Australian tweets expressed anger toward politicians who were perceived to delay restrictions on mass gatherings based on their support for specific sporting teams.

Discussion about cultural events included religious gatherings. Tweets from Ireland displayed disbelief at the cancellation of Catholic Mass but quickly embraced the use of live streaming of services. Canadian tweets expressed solidarity and support for those celebrating religious holidays. Conversely, US tweets demonstrated anger toward specific religious figures or groups who continued to gather in large groups.

Irish and US tweets discussed the cancellation of St. Patrick’s Day (March 17, 2020) festivities. Interestingly, the proportion of Irish tweets, which uniformly demanded the cancellation of festivities, were less than those seen in the US data set where the response was mixed. The cancellation of Australia’s ANZAC day services (April 25, 2020) had a sobering effect, and while many encouraged the observation of services at home, an attitudinal shift was noted regarding the seriousness of the situation.

The US NPI topics on gatherings in public places accounted for 30.42% (23,099/75,938) of NPI-related tweets, the most intense of all NPIs. The majority of tweets passionately debated the implications for the 2020 US general election. The US public perceived the then-current campaign schedule as a threat to community health and urged postponement or postal voting to avoid increased transmission. Compulsory in-person voting in the Australian Queensland by-election (March 28, 2020) attracted scathing commentary from those who perceived the directive to be inconsistent with social distancing mandates.

Media coverage of beachgoers failing to socially distance in Florida and Sydney saw US and Australian tweets calling for beach closures. Additionally, Australian and Canadian tweets called for closures of running paths if social distancing did not improve.

### Lockdowns

Five NPIs pertained to Lockdowns: Public Support, High-Risk Groups Vulnerable Groups, Self-Care, and Legal Enforcement.

There was broad support for lockdowns across countries. Before the implementation of national lockdowns in Australia, Ireland, and the United Kingdom, the public showed increasing frustration with the delayed implementation of these lockdowns. UK tweets called for tighter restrictions as a means of deterring noncompliant behaviors and protecting the NHS from being overwhelmed. Earlier Australian tweets, comparing their incremental introduction of restrictions to the New Zealand lockdown [34], indicated a preference for a complete lockdown. New Zealand tweets were calm and accepting. “Be kind” was consistently seen in these tweets, reflecting the New Zealand government’s “Unite against COVID-19” campaign messages [35].

The message to stay at home was promoted in the US and UK data sets with a sense of urgency. These tweets pleaded for people to “stay home and save lives.” UK tweets posted before the implementation of a lockdown perceived staying home as a civic duty. Australian tweets encouraged people to stay at home, but acknowledged people needed some flexibility to do so.

A large proportion of UK tweets expressed concern for high-risk community members (64,063/219,485, 29.19%), such as the elderly, and their protection was seen to be a community responsibility. Both UK and Australian tweets applauded grocery chains that reserved times for elderly or disabled individuals to make purchases. Discussion of outbreaks in aged care facilities differed between countries. Canadians blamed the outbreaks on inadequate PPE and cross-facility rostering of the staff, whereas Australian tweets blamed staff who attended work when ill, and Irish tweets blamed the broader public for not adhering to restrictions. Both the Irish and US tweets reported sadness and disappointment that their efforts to quarantine high-risk family members in aged care homes were ineffective.

The proportion of New Zealand tweets about vulnerable groups was the largest of all data sets (10,844/47,500, 22.83%). The discussion was mainly about the protection of the Māori people from the pandemic, which was perceived by the public as a national responsibility and called for culturally appropriate response plans.

The vulnerability of individuals in prison and immigration detention was discussed in all data sets except New Zealand. UK tweets appeared sympathetic to those in prison but also perceived early prison release programs as a risk to public safety. Canadian tweets supported only the release of those on remand for nonviolent crimes. US tweets did not agree with early prison release programs. Australian, UK, Canadian and Irish tweets aimed to raise awareness of the vulnerability of both refugees and those in immigration detention. Canadian and Irish tweets discussed the risk of transmission in homeless and women's shelters and amongst itinerant communities. The requisitioning of hotels to facilitate self-isolation and increased hygiene was supported.

Aside from the United States, tweets from all countries demonstrated a positive attitude toward staying well and discussed the importance of self-care, nutrition, routines, social connection, and exercise. Australian tweets self-reported mental health symptoms but indicated proactive management. In contrast, pessimistic UK tweets discussed the lack of funding for mental health services.

Attitudes toward increased police and military powers to enforce restrictions were perceived differently within the Irish data set. While the majority supported increased police presence in public, their role in enforcing lockdown received some criticism. Similarly, there was debate over a recent challenge in the Irish high court over the enforcement of these restrictions. The Australian mainstream media labeled the legal enforcement of restrictions as draconian. However, this perception was not consistent with the public, who mostly supported legal enforcement. The exception, however, was for the issuing of “frivolous” noncompliance fines, which were perceived as overzealous and unnecessary. New Zealand tweets demonstrated a positive attitude toward police and their approach to enforcement. UK tweets were mainly supportive of military enforcement and anxious to see these measures undertaken.

### Travel Restrictions

Three NPIs about Travel Restrictions were identified: Travel Bans, Travel Quarantine, and Border Closures.

New Zealand tweets were highly supportive of the government's decision to place a travel ban on arrivals from mainland China who were not residents of New Zealand. Referencing New Zealand’s travel ban, UK and Irish tweets were outraged that similar measures had not yet been enacted.

US tweets called for increased quarantine measures for cruise ships to the standard applied to air travel. Australian tweets displayed outrage that passengers of the Ruby Princess cruise ship, from which 22 deaths and 700 cases were reported [36], were told only to self-quarantine. Both the Australian and Canadian public lacked confidence in people’s adherence to self-quarantine rules. Australian tweets supported the forcible quarantining of return-travelers, although many asked for further clarification about this decision.

Australian tweets supported the closure of international borders [37] but discussed difficulties for Australian residents returning home. Both Australian and US tweets called for internal border closures, praising leaders who took this measure. US and Canadian tweets called for the US-Canadian border to be closed due to the rapid rise in US cases.

### Workplace Closures

Three NPIs about Workplace Closures were identified: Nonessential Services, School Closures, and Working at Home.

Australian, Irish, UK, and US tweets called for the closure of nonessential businesses. Australian and UK tweets reasoned that keeping businesses open encouraged noncompliant behaviors. Irish tweets perceived the closure of schools to be illogical when the hospitality industry remained open.

Parents concerned about the health of their children accounted for the majority of Australian, Canadian, Irish, and UK tweets about school closures. Homeschooling and curriculum continuity were discussed, with many seeking advice about accessing online learning materials, and the implications for high-school and university exams. Australian tweets were frustrated by the discrepancies between federal and state positions on school closures and the contradictory health advice about transmission in children.

Australian tweets highlighted the positive aspects of working at home and expressed surprise at the ease of transition to online meetings. Canadian tweets were contemplative of the adaptions but embraced their new arrangements as time progressed.

## Discussion

### Principal Findings

In this study, we found that Twitter offers a means by which governments and health authorities can gain rapid feedback about public perceptions and attitudes on NPIs. Topic modeling was used to identify seven categories of NPIs discussed in tweets from the six selected countries. A comparative analysis of NPI topics showed that less restrictive NPIs were broadly supported in all data sets with much of the public encouraging adherence to these restrictions. However, public attitudes toward restrictive NPIs differed between countries.

Four characteristics of NPI regimes were common to all countries and identified as potential predictors of public adherence to NPIs: timeliness of implementation, NPI campaign strategies, inconsistent information, and enforcement strategies.

The timeliness of the implementation of restrictive NPIs influenced public attitudes. Prolonged, staggered, or delayed implementation of restrictive NPIs was met by an angry and fearful public response and demands for restrictions to be increased, as seen in Australian, UK, and Irish tweets. Conversely, tweets from New Zealand, where there was a sudden and total implementation of highly restrictive NPIs, including lockdowns and workplace closures, showed overwhelming support for the regime when enacted despite the low number of cases. These observations indicate that delayed implementation of restrictions may heighten the public’s sense of uncertainty about potentially increased restrictions.

Uncertainty may also be a result of inconsistencies between government recommendations, medical expertise, and global health organizations’ advice. Information about NPIs not offered by governments was shared broadly on Twitter. For example, many tweets discouraged face touching despite there being limited government messaging regarding this behavior across the six countries. Information asymmetry and inconsistency had a greater effect when NPIs were more intrusive. We observed that Australian and UK public perceptions of face masks were inconsistent with government advice. Public attitudes toward face masks were mixed. Despite them not being recommended by either country’s governments at the time, they were mainly regarded as appropriate by the public. However, many debated their necessity and efficacy, often supporting their position with scientific literature, references to other governments, health organizations’ recommendations, and media articles. The public’s access to alternative information via Twitter may impact their confidence in national NPI regimes when it conflicts with advice from government and health bodies.

Another influencing characteristic was the style of government NPI campaign strategies. Both the UK and New Zealand governments adopted strategies that fostered a strong sense of collective action. A key point of difference was the emphasis on unity, clarity, and empathy, as seen in New Zealand’s “Unite against COVID-19” campaign with the public expressing positive attitudes toward NPIs. Campaign messages were fashioned into popular hashtags, including #BeKind, #BreaktheChain, and #StayInYourBubble. Significantly, New Zealand tweets emphasized empathy and kindness, a key element of the government's campaign strategy.

Conversely, the UK government adopted an instructive campaign to “stay home, protect the NHS, save lives.” The public perceived it to be their collective responsibility to protect health care workers and those most at risk. However, the delayed implementation of more restrictive NPIs resulted in negative attitudes toward the government. These results indicate that effect- and emotion-based campaigns are potentially more compelling in maintaining adherence to NPIs, but their success is reliant on other factors.

The enforcement of social distancing and lockdowns appeared not to impact the public attitudes toward restrictions. This may be because there was already overwhelming support for these measures. Compliance in the United Kingdom has been linked to people’s intrinsic motivation to obey the law [38]. However, we observed that noncompliant behaviors were unintentional and a result of a misunderstanding of the rules. As suggested by previous studies [7], these findings suggest that the ambiguous and dynamic nature of NPIs and the way they are communicated were factors that contributed to comprehension.

### Strengths and Limitations

This study has a number of limitations. First, the demographics of Twitter users may not necessarily represent the populations of each of the selected countries [39]. Second, the choice to include only English tweets means that nonanglophones were not represented. However, our methods are consistent with medical research that makes use of topic modeling of tweets and is often restricted to the official language of the country [39]. Furthermore, we expand upon previous approaches by undertaking a structured qualitative analysis of topic document collections, which are further contextualized through the review of related hypermedia. This hybrid approach provides the depth of insight offered by qualitative methods, with the speed and scalability of computational techniques.

### Conclusions

The effectiveness of COVID-19 NPI regimes is dependent on ongoing public adherence. Given this, it is necessary to understand public perceptions and attitudinal trends toward these NPIs, as well as why and in what contexts these behaviors arise and persist [6]. As such, our study was motivated by the need to inform an understanding of such trends to support government communication strategies, as well as the planning and implementation of more effective NPI regimes in later phases of the COVID-19 pandemic.

We undertook a hybrid computational analysis of the public discussion of COVID-19 NPIs across six countries. As detailed above, four characteristics of NPIs regimes were identified as potential predictors of public adherence. The outcome of our analysis is that the widespread public acceptance of NPI regimes is predicated on the public’s understanding, timeliness of implementation, the ability of governments to clearly communicate and justify the complexity of the regime, and importantly, their ability to implement the regime without ambiguity or undue enforcement measures.

Ongoing analysis of social media offers governments and health authorities insights into how their programs are heard as well as a critical perspective of their communication strategies. Such feedback should be integrated to produce a more effective public health response to the ongoing pandemic as well as future disease outbreaks. Ongoing and expanded analyses of social media will contribute to a richer understanding of attitudinal and behavioral drivers to inform public health strategies.

## Appendix

Multimedia Appendix 1 Hashtags used for tweet retrieval.

Multimedia Appendix 2 Graphical analysis of the frequency of tweets per day against the number of confirmed cases per day, and chord diagrams of NPI topic category tweet co-occurrences.

Multimedia Appendix 3 Results of the Pearson correlation analysis of the relationship between the number of confirmed cases and tweets per day.

Multimedia Appendix 4 NPI topic word sets, topic model evaluations, and the frequency of topics per NPI for each country.

Multimedia Appendix 5 NPI topic coding schema.

Multimedia Appendix 6 Exemplar tweets that illustrate the public discussion of NPIs.

## Abbreviations

API: application programming interface
LDA: Latent Dirichlet Allocation
NHS: National Health Service
NPI: nonpharmaceutical intervention
NPMI: normalized pointwise mutual information
PPE: personal protective equipment
RTP: Research Training Program
WHO: World Health Organization
