# Supplementary material for: Public Perceptions and Attitudes Toward COVID-19 Nonpharmaceutical Interventions Across Six Countries: A Topic Modeling Analysis of Twitter Data
Source: J Med Internet Res. 2020 Sep 3;22(9):e21419. doi: 10.2196/21419 (PMC7505256; doi:10.2196/21419)
Supplement: Multimedia Appendix 1 [file jmir_v22i9e21419_app1.docx]

**Multimedia Appendix 1. Hashtags used for tweet retrieval**

| **Data set** | **Tweets (n)** |
| --- | --- |
| Australia | 243,687 |
| Canada | 94399 |
| Ireland | 50,862 |
| New Zealand | 53,311 |
| United Kingdom | 236,899 |
| United States | 98,711 |
| ***Total*** | 777,869 |

**Table 1.** **The number (n) of tweets analysed for each country.** The number of tweets in each dataset that were included in the analysis after pre-processing.

| **Australia** | **Canada** | **Ireland** |
| --- | --- | --- |
| **Primary hashtags** | | |
| #covid19au | #covid19canada | #coronaireland |
| #coronavirusaus | #covidcanada | #coronavirusireland |
| #covidaustralia | #coronaviruscanada | #covid19ireland |
| **Secondary hashtags** | | |
| #lockdownaustralia | #coronavirustoronto | # covid_19ireland |
| #coronaaustralia | #covidalberta | #irelandlockdown |
| #coronavirusau | #coronavirustoronto | #irelandvscovid |
| #covid_19australia | #covid19on | #lockdownireland |
| #coronavirusaustralia | #covid19ontario |  |
| #covid19aus | #covid19toronto |  |
| #covid19australia | #covidbc |  |
| #covid2019au | #covidontario |  |
| #lockdownaustralia | #covidtoronto |  |
| #shutdownaustralia | #stayhomecanada |  |
| #stayhomeaustralia | #covid19bc |  |

**Table 2.** **Tweet retrieval hashtags for Australia, Canada, and Ireland.** Primary hashtags are used to seed the retrieval of tweets. Secondary hashtags are derived from the initial collection and used to extend the collection of tweets.

| **New Zealand** | **United Kingdom** | **United States** |
| --- | --- | --- |
| **Primary hashtags** | | |
| #covid19nz | #covid19uk | #coronavirususa |
| #coronoavirusnz | #coronaviursuk | #covid19usa |
| #nzlockdown | #londonlockdown | #covidus |
| **Secondary hashtags** | | |
| #closenz | #uklockdown | #coronavirusflorida |
| #closenz | #covid_19uk | #coronavirusnewyork |
| #covid_19nz | #covidー19uk | #coronavirusnyc |
| #covidnz | #covid2019uk | #coronavirusus |
| #lockdownnz |  | #covid19us |
| #newzealandlockdown |  | #covidusa |
| #stayhomenz |  | #usacoronavirus |
| #stayinyourbubble |  | #coronavirusseattle |

**Table 3.** **Tweet retrieval hashtags for New Zealand, the United Kingdom, and the United States.** Primary hashtags are used to seed the retrieval of tweets. Secondary hashtags are derived from the initial collection and used to extend the collection of tweets. Tweets from the united states did not yield enough country specific secondary hashtags. Secondary hashtags were drawn from within the top 500 hashtags.

| **Rank** | **Australia** | **n** | **Canada** | **n** |
| --- | --- | --- | --- | --- |
| **1** | #covid19australia | 33426 | #covid19 | 14319 |
| **2** | #auspol | 30387 | #cdnpoli | 8431 |
| **3** | #covid19aus | 5982 | #covidー19 | 3383 |
| **4** | #covidー19 | 5982 | #onpoli | 3075 |
| **5** | #covid19 | 5406 | #ableg | 3003 |
| **6** | #scottyfrommarketing | 4095 | #covid_19 | 2896 |
| **7** | #covid_19australia | 2921 | #covidbc | 2768 |
| **8** | #coronavirusaus | 2913 | #socialdistancing | 1822 |
| **9** | #coronaaustralia | 2240 | #covidontario | 1818 |
| **10** | #covid_19 | 2183 | #yeg | 1607 |
| **11** | #lockdownaustralia | 2095 | #yyc | 1567 |
| **12** | #stayathome | 1680 | #covid2019 | 1559 |
| **13** | #socialdistancing | 1614 | #flattenthecurve | 1496 |
| **14** | #coronavirusau | 1530 | #bcpoli | 1478 |
| **15** | #covid2019au | 1519 | #coronavirustoronto | 1473 |
| **16** | #covid2019 | 1432 | #covid19ontario | 1410 |
| **17** | #flattenthecurve | 1403 | #coronavirusoutbreak | 1256 |
| **18** | #rubyprincess | 1337 | #abpoli | 1173 |
| **19** | #scomo | 1232 | #stayhome | 1125 |
| **20** | #insiders | 1149 | #pandemic | 840 |
| **21** | #nswpol | 1064 | #toronto | 839 |
| **22** | #lockdown | ~~915~~ | #coronavirusupdate | 809 |
| **23** | #auspoi | 915 | #cdnmedia | 785 |
| **24** | #shutdownaustralia | 908 | #covid19bc | 762 |
| **25** | #coronavirusoutbreak | 821 | #coronaviruspandemic | 744 |
| **26** | #stayhome | 789 | #covidtoronto | 738 |
| **27** | #auspol2020 | 759 | #canlab | 736 |
| **28** | #covidiots | 742 | #stayathome | 709 |
| **29** | #qanda | 737 | #abhealth | 690 |
| **30** | #closetheschools | 658 | #ontario | 605 |
| **31** | #shuttheschools | 657 | #covid19toronto | 544 |
| **32** | #stayhomeaustralia | 651 | #covid19on | 540 |
| **33** | #centrelink | 639 | #stayhomesavelives | 524 |
| **34** | #springst | 636 | #physicaldistancing | 519 |
| **35** | #scottyfommarketing | 630 | #trudeau | 496 |
| **36** | #lnp | 620 | #alberta | 483 |
| **37** | #lnpfail | 617 | #stayhomecanada | 474 |
| **38** | #qldpol | 601 | #canadacovid19 | 437 |
| **39** | #stayhomesavelives | 542 | #yql | 423 |
| **40** | #scottmorrison | 511 | #vancouver | 422 |
| **41** | #breaking | 502 | #canpoli | 411 |
| **42** | #lockusdown | 497 | #cdnhealth | 405 |
| **43** | #nrl | 496 | #coronaoutbreak | 399 |
| **44** | #coronalockdown | 487 | #quarantinelife | 389 |
| **45** | #ausbiz | 484 | #ottawa | 386 |
| **46** | #abcnews | 482 | #staysafestayhome | 384 |
| **47** | #covidsafe | 476 | #selfisolation | 382 |
| **48** | #sydney | 475 | #topoli | 372 |
| **49** | #abc730 | 460 | #covidiots | 367 |
| **50** | #selfisolation | 445 | #cerb | 365 |

**Table 4.** **Top 50 ranked hashtags for Australia and Canada.** Hashtags are ranked in order of highest frequency (n). Primary hashtags are excluded from the analysis.

| **Rank** | **Ireland** | **n** | **New Zealand** | **n** |
| --- | --- | --- | --- | --- |
| **1** | #covid19 | 4095 | #covid19nz | 11466 |
| **2** | #covidー19 | 2132 | #nzpol | 3045 |
| **3** | #covid_19 | 8203 | #covid19 | 2439 |
| **4** | #covid2019 | 2132 | #covid_19nz | 1992 |
| **5** | #socialdistancing | 1525 | #covid_19 | 1228 |
| **6** | #stayhome | 1068 | #lockdownnz | 1085 |
| **7** | #stayathome | 955 | #newzealand | 911 |
| **8** | #irelandlockdown | 861 | #stayhomenz | 701 |
| **9** | #vmnews | 825 | #covidー19 | 409 |
| **10** | #flattenthecurve | 759 | #lockdown | 377 |
| **11** | #lockdownireland | 665 | #stayhome | 304 |
| **12** | #irelandvscovid | 527 | #auspol | 259 |
| **13** | #coronaviruspandemic | 509 | #newzealandlockdown | 255 |
| **14** | #lockdown | 490 | #coronavirusnz | 254 |
| **15** | #stayhomesavelives | 481 | #covid2019 | 244 |
| **16** | #dublin | 476 | #jacindaardern | 231 |
| **17** | #coronavirusuk | 448 | #bekind | 173 |
| **18** | #covid2019ireland | 434 | #stayhomesavelives | 163 |
| **19** | #hse | 423 | #stayathome | 154 |
| **20** | #stpatricksday | 410 | #staysafe | 152 |
| **21** | #covid19uk | 409 | #auckland | 144 |
| **22** | #covidireland | 409 | #covid19aus | 126 |
| **23** | #coronavirusoutbreak | 405 | #coronaviruslockdown | 122 |
| **24** | #coronavirusupdate | 392 | #level3 | 122 |
| **25** | #staysafe | 387 | #selfisolation | 120 |
| **26** | #coronalockdown | 357 | #uniteagainstcovid19 | 118 |
| **27** | #covid_19ireland | 347 | #covid19australia | 109 |
| **28** | #cork | 318 | #closenz | 97 |
| **29** | #rtept | 316 | #covid_19australia | 97 |
| **30** | #coronavirusupdates | 301 | #coronavirusoutbreak | 97 |
| **31** | #selfisolation | 294 | #flattenthecurve | 95 |
| **32** | #cblive | 289 | #wellington | 94 |
| **33** | #closethepubs | 252 | #covid19au | 90 |
| **34** | #staysafestayhome | 248 | #pandemic | 90 |
| **35** | #tonightvmtv | 239 | #level4 | 86 |
| **36** | #washyourhands | 227 | #covidnz | 85 |
| **37** | #inthistogether | 217 | #coronalockdown | 85 |
| **38** | #stayathomeandstaysafe | 215 | #socialdistancing | 80 |
| **39** | #coronvirusireland | 210 | #kiakaha | 79 |
| **40** | #ppe | 206 | #breaking | 79 |
| **41** | #coronaoutbreak | 197 | #covidcollate | 79 |
| **42** | #coronacrisis | 193 | #australia | 77 |
| **43** | #stayathomesavelives | 183 | #coronavirusaustralia | 71 |
| **44** | #workingfromhome | 170 | #news | 65 |
| **45** | #leovaradkar | 166 | #covid19uk | 63 |
| **46** | #covid19pandemic | 165 | #workingfromhome | 60 |
| **47** | #wexford | 162 | #coronavirusupdate | 60 |
| **48** | #todaysor | 153 | #stayinyourbubble | 59 |
| **49** | #covidiots | 142 | #isolation | 56 |
| **50** | #liveline | 139 | #lockdown20202 | 54 |

**Table 5.** **Top 50 ranked hashtags for Ireland and New Zealand.** Hashtags are ranked in order of highest frequency (n). Primary hashtags are excluded from the analysis.

| **Rank** | **United Kingdom** | **n** | **United States** | **n** |
| --- | --- | --- | --- | --- |
| **1** | #covid19uk | 81884 | #covid19 | 16586 |
| **2** | #covid19 | 25428 | #coronavirusnewyork | 6891 |
| **3** | #covid_19uk | 12185 | #covidー19 | 6480 |
| **4** | #nhs | 7926 | #coronavirusoutbreak | 6146 |
| **5** | #covid_19 | 7432 | #covid_19 | 4733 |
| **6** | #covidー19 | 6568 | #coronaviruspandemic | 4105 |
| **7** | #covid2019 | 4374 | #covid2019 | 3806 |
| **8** | #coronavirusoutbreak | 4108 | #coronavirusupdate | 2989 |
| **9** | #borisjohnson | 3269 | #trump | 2821 |
| **10** | #lockdown | 3185 | #coronavirusupdates | 2358 |
| **11** | #stayhomesavelives | 3183 | #coronaoutbreak | 1741 |
| **12** | #lockdownuk | 2489 | #pandemic | 1643 |
| **13** | #coronavirusupdate | 2484 | #covid19us | 1338 |
| **14** | #coronaviruspandemic | 2473 | #china | 1237 |
| **15** | #socialdistancing | 2151 | #stayhome | 1189 |
| **16** | #stayathome | 2025 | #trumpvirus | 1065 |
| **17** | #covidー19uk | 1954 | #coronavirustruth | 937 |
| **18** | #selfisolation | 1940 | #maga | 934 |
| **19** | #uklockdown | 1886 | #socialdistancing | 890 |
| **20** | #stayhome | 1713 | #newyork | 788 |
| **21** | #nhsheroes | 1647 | #covid19pandemic | 775 |
| **22** | #brexit | 1563 | #stayathome | 755 |
| **23** | #coronacrisisuk | 1533 | #cdc | 753 |
| **24** | #ppe | 1531 | #sarscov2 | 737 |
| **25** | #staysafe | 1524 | #coronavirusuk | 690 |
| **26** | #coronauk | 1316 | #coronaupdate | 673 |
| **27** | #london | 1238 | #coronalockdown | 602 |
| **28** | #coronaoutbreak | 1232 | #wuhan | 567 |
| **29** | #stayathomesavelives | 1217 | #coronavid19 | 541 |
| **30** | #coronacrisis | 1147 | #coronaviruslockdown | 540 |
| **31** | #coronaviruslockdown | 1138 | #flattenthecurve | 524 |
| **32** | #coronapocolypse | 1101 | #wuhanvirus | 506 |
| **33** | #covid2019uk | 1040 | #lockdown | 494 |
| **34** | #coronavirusupdates | 1022 | #flu | 476 |
| **35** | #bbcnews | 1021 | #quarantine | 474 |
| **36** | #schoolclosuresuk | 999 | #covidiots | 443 |
| **37** | #covidiots | 998 | #covid19uk | 439 |
| **38** | #herdimmunity | 931 | #italy | 435 |
| **39** | #mentalhealth | 927 | #quarantinelife | 432 |
| **40** | #workingfromhome | 900 | #donaldtrump | 397 |
| **41** | #panicbuying | 885 | #kag | 392 |
| **42** | #schoolclosure | 848 | #2019ncov | 388 |
| **43** | #pandemic | 847 | #nyc | 385 |
| **44** | #coronaviruschallenge | 832 | #potus | 368 |
| **45** | #borisout | 817 | #coronavirusindia | 364 |
| **46** | #selfisolating | 806 | #coronapocolypse | 344 |
| **47** | #coronaviruslockdown | 776 | #breaking | 339 |
| **48** | #dailybriefing | 768 | #covidiot | 333 |
| **49** | #borisresign | 727 | #trumppandemic | 327 |
| **50** | #keyworkers | 171 | #stayhomesavelives | 323 |

**Table 6.** **Top 50 ranked hashtags for the United Kingdom and the United States.** Hashtags are ranked in order of highest frequency (n). Primary hashtags are excluded from the analysis.
