# Supplementary material for: Public Perceptions and Attitudes Toward COVID-19 Nonpharmaceutical Interventions Across Six Countries: A Topic Modeling Analysis of Twitter Data
Source: J Med Internet Res. 2020 Sep 3;22(9):e21419. doi: 10.2196/21419 (PMC7505256; doi:10.2196/21419)
Supplement: Multimedia Appendix 2 [file jmir_v22i9e21419_app2.docx]

**Multimedia Appendix 2. Graphical Analysis of the frequency of tweets per day against the number of confirmed cases per day, and Chord diagrams of NPI topic category tweet co-occurrences.**

Frequency of tweets per day against the number of confirmed cases per day (scaled).

**
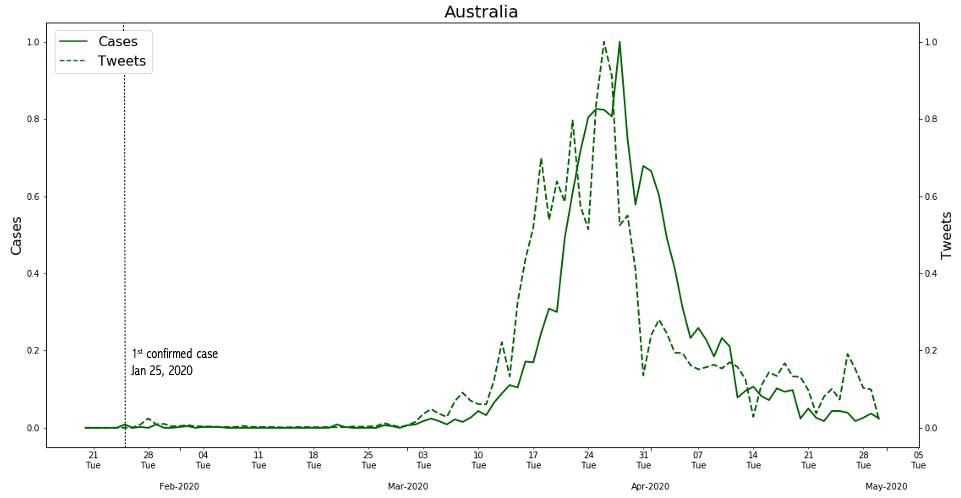
**

**Figure 1. Tweets and confirmed cases per day for Australia.**

**
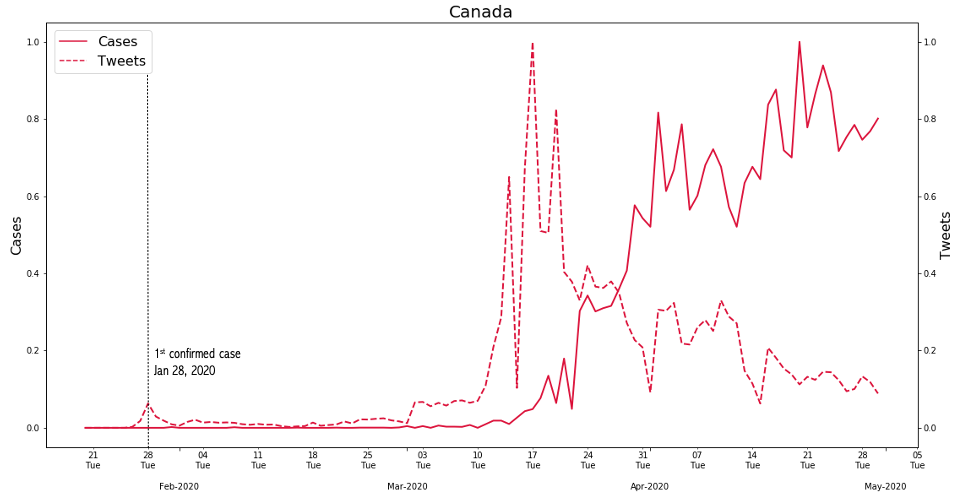
**

**Figure 2. Tweets and confirmed cases per day for Canada.**

**
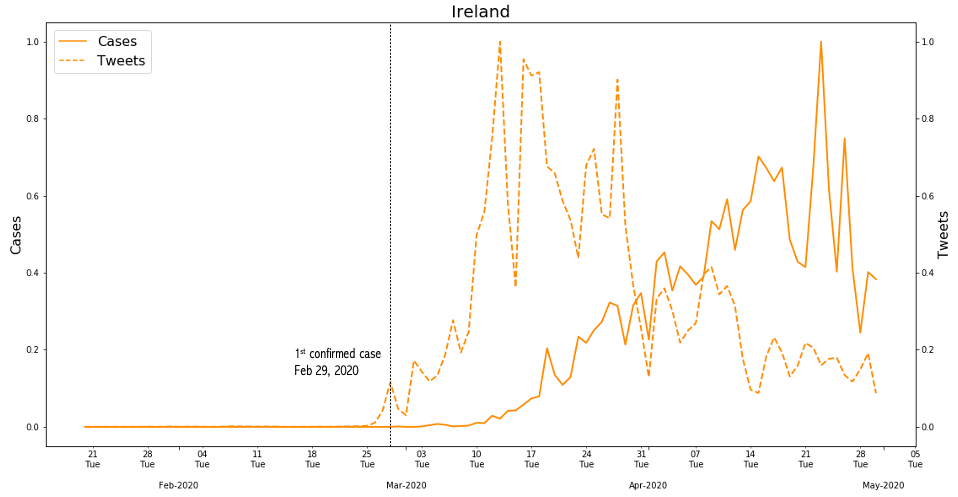
**

**Figure 3. Tweets and confirmed cases per day for Ireland.**

**
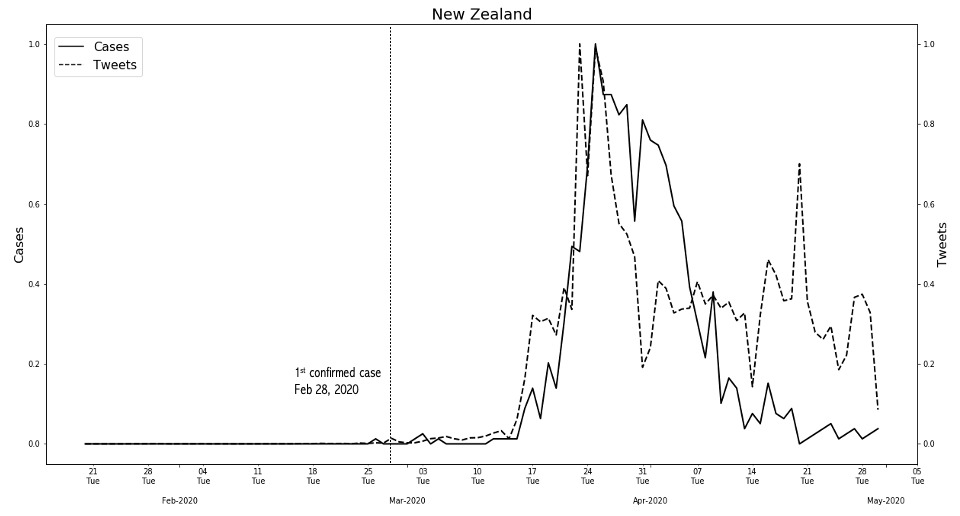
**

**Figure 4. Tweets and confirmed cases per day for New Zealand.**

**
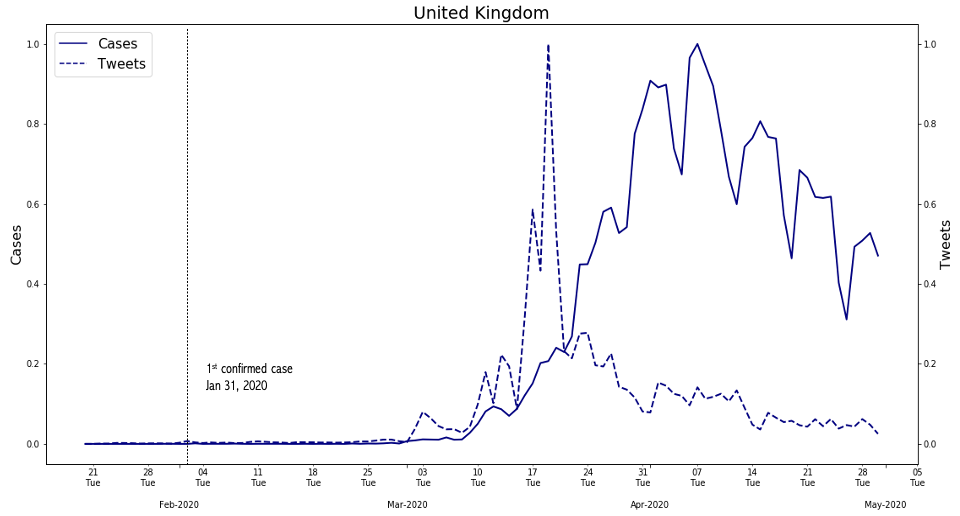
**

**Figure 5. Tweets and confirmed cases per day for the United Kingdom.**

**
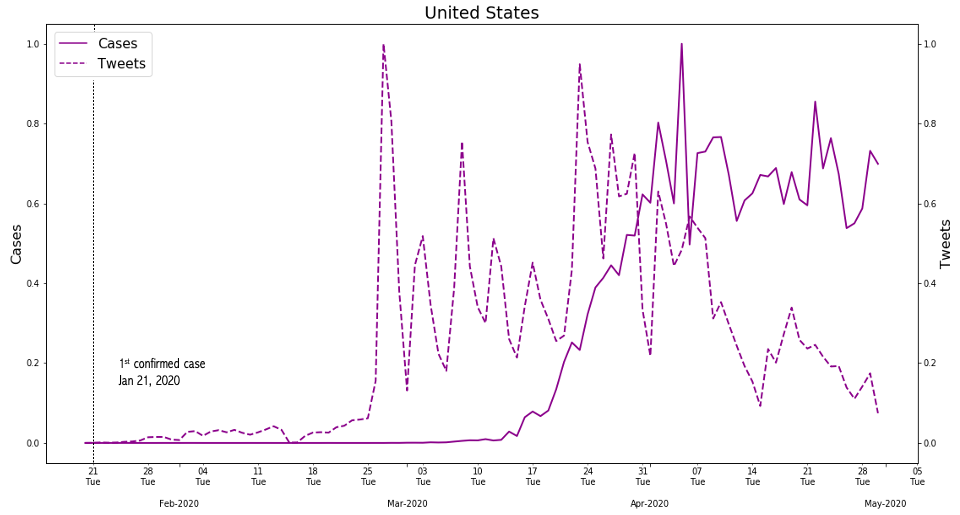
**

**Figure 6. Tweets and confirmed cases per day for the United States.**

Chord diagrams of NPI topic category tweet co-occurrences.

**
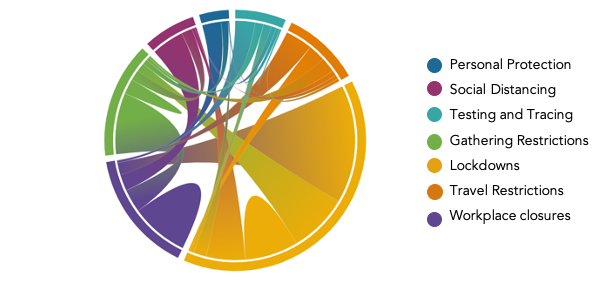
**

**Figure 7. A Chord diagram showing the relationship between NPI categories for Australia.**

**
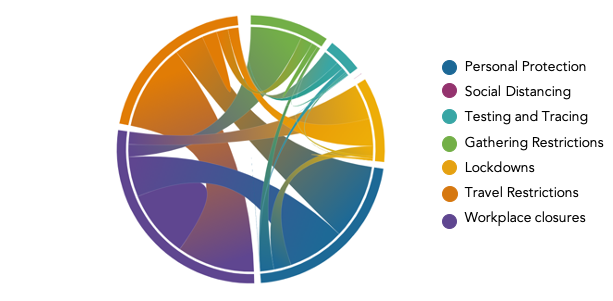
**

**Figure 8. A Chord diagram showing the relationship between NPI categories for Canada.**

**
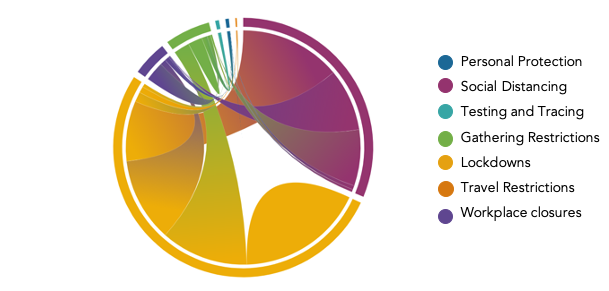
**

**Figure 9. A Chord diagram showing the relationship between NPI categories for Ireland.**

**
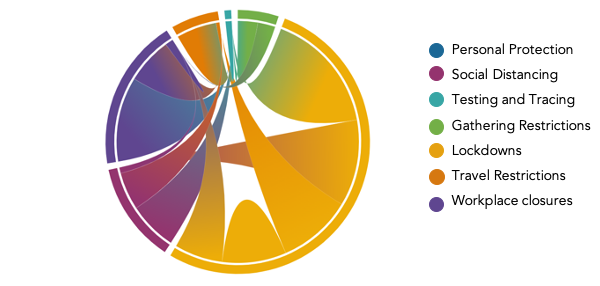
**

**Figure 10. A Chord diagram showing the relationship between NPI categories for New Zealand.**

**
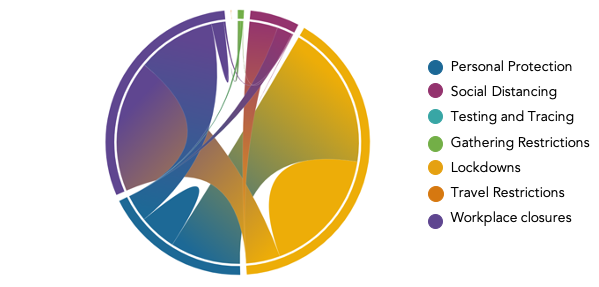
**

**Figure 11. A Chord diagram showing the relationship between NPI categories for the United Kingdom.**

**
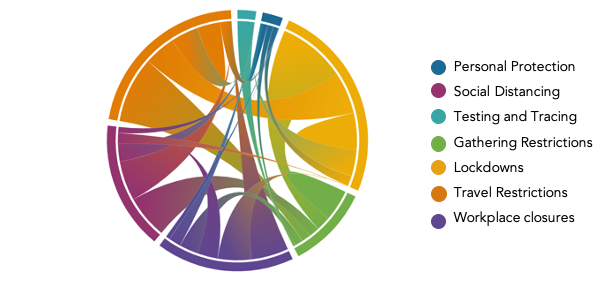
**

**Figure 12. A Chord diagram showing the relationship between NPI categories for the United States.**
