# Supplementary material for: Public Perceptions and Attitudes Toward COVID-19 Nonpharmaceutical Interventions Across Six Countries: A Topic Modeling Analysis of Twitter Data
Source: J Med Internet Res. 2020 Sep 3;22(9):e21419. doi: 10.2196/21419 (PMC7505256; doi:10.2196/21419)
Supplement: Multimedia Appendix 3 [file jmir_v22i9e21419_app3.docx]

**Multimedia Appendix 3. Results of the Pearson correlation analysis of the relationship between the number of confirmed cases and tweets per day**

| **Time periods** | **Australia** | | **Canada** | | **Ireland** | |
| --- | --- | --- | --- | --- | --- | --- |
| Date. 1st confirmed case | January 25, 2020 | | January 31, 2020 | | 29 February 2020 | |
| No. days since 1st case (n) | 97 | | 91 | | 62 | |
|  | ***r*** | ***P*** | ***r*** | ***P*** | ***r*** | ***P*** |
| **No. Tweets (per day)** | | | | | | |
| All days (N=121) | 0.837 | < .001 | 0.299 | < .001 | 0.262 | 0.004 |
| Since 1st case (n) | 0.823 | < .001 | 0.174 | 0.099 | -0.363 | 0.004 |
| **No. tweets (3-day moving average)** | | | | | | |
| All days (N=121) | 0.801 | < .001 | 0.287 | < .001 | 0.245 | 0.007 |
| Since 1st case (n) | 0.784 | < .001 | 0.162 | 0.126 | -0.408 | < .001 |

**Table 1. Pearson correlation coefficients (*r*) for the relation of the frequency of tweets with the number of confirmed cases of COVID-19 for Australia, Canada, and Ireland.** The number of tweets is calculated as a daily frequency or a 3-day moving average. The calculation is either conducted for the full period of time surveyed (January 1, 2020 to April 30, 2020, N=121), or for the number of days since the first confirmed case of COVID-19 was reported (n).

| **Time periods** | **New Zealand** | | **United Kingdom** | | **United States** | |
| --- | --- | --- | --- | --- | --- | --- |
| Date. 1st confirmed case | February 28, 2020 | | January 30, 2020 | | 21 January 2020 | |
| No. days since 1st case (n) | 65 | | 92 | | 101 | |
|  | ***r*** | ***P*** | ***r*** | ***P*** | ***r*** | ***P*** |
| **No. Tweets (per day)** | | | | | | |
| All days (N=121) | 0.747 | < .001 | 0.273 | 0.002 | 0.375 | < .001 |
| Since 1st case (n) | 0.666 | < .001 | 0.170 | 0.105 | 0.312 | < .001 |
| **No. tweets (3-day moving average)** | | | | | | |
| All days (N=121) | 0.732 | < .001 | 0.250 | 0.006 | 0.379 | < .001 |
| Since 1st case (n) | 0.641 | < .001 | 0.145 | 0.167 | 0.295 | 0.003 |

**Table 2. Pearson correlation coefficients (*r*) for the relation of the frequency of tweets with the number of confirmed cases of COVID-19 for New Zealand, the United Kingdom, and the United States.** The number of tweets is calculated as a daily frequency or a 3-day moving average. The calculation is either conducted for the full period of time surveyed (January 1, 2020 to April 30, 2020, N=121), or for the number of days since the first confirmed case of COVID-19 was reported (n).
