# Supplementary material for: Public Perceptions and Attitudes Toward COVID-19 Nonpharmaceutical Interventions Across Six Countries: A Topic Modeling Analysis of Twitter Data
Source: J Med Internet Res. 2020 Sep 3;22(9):e21419. doi: 10.2196/21419 (PMC7505256; doi:10.2196/21419)
Supplement: Multimedia Appendix 4 [file jmir_v22i9e21419_app4.docx]

**Multimedia Appendix 4. NPI topic word sets, topic model evaluations, and the frequency of topics per NPI for each country**

| **Topic** | **Topic word set** |
| --- | --- |
| 3 | test, symptom, doctor, day, tell, influenza, cough, contact, result, fever |
| 4 | ship, cruise ship, passenger, ruby princess, allow, sydney, dock, disembark, crew, infection |
| 10 | sydney, city, melbourne, new_york, lock_down, centre, airport, major, cuomo, march |
| 12 | close, border, island, south, come, country, tasmania, nation, north, eastern |
| 15 | stay, home, safe, work, keep, please, social distance, live, stop, help |
| 17 | hair dresser, hair, cut, minute, minimum, haircut, open, time, long, rule |
| 23 | lock down, measure, spread, government, restriction, week, down, control, month, slow |
| 24 | community, risk, family, sign, women, refugee, prison, release, aboriginal, right |
| 29 | shop, supermarket, delivery, food, store, local, customer, buy, staff, order |
| 30 | essential, shop, open, service, centre, close, home, food, funeral, stay |
| 32 | flight, travel, international, cancel, airline, home, sydney, fly, domestic, due |
| 38 | close, shut, down, school, open, pub, restaurant, business, club, cafe |
| 40 | app, data, government, track, download, privacy, trace, contact trace, use, phone |
| 51 | day, per, one, week, anzac, hour, ago, time, take, person |
| 53 | time, feel, social, help, anxiety, isolate, stress, fear, mental health, change |
| 54 | train, bus, car, social distance, drive, public transport, line, stand, sit, hour |
| 60 | beach, park, walk, social distance, close, sydney, today, street, car, local |
| 61 | court, law, legal, lawyer, under, change, trial, pas, hear, victoria |
| 62 | test, case, number, positive, community, symptom, contact, criterion, infection, result |
| 71 | mask, supply, china, medical, ppe, ventilator, manufacture, government, make, test kit |
| 72 | quarantine, arrive, travel, self isolate, day, hotel, return, overseas, isolate, border |
| 73 | cancel, event, football, weekend, sport, due, conference, ahead, postpone, game |
| 75 | state, victoria, nsw, premier, health, government, close, minister, queensland, announce |
| 77 | police, fine, law, rule, nsw, social distance, break, state, issue, victoria |
| 78 | Social distance, rule, gather, school, distance, measure, person, ban, one, limit |
| 79 | mask, wear, face, hand, touch, wash hand, clean, cough, glove, wash |
| 82 | parliament, vote, government, queensland, election, sit, federal, local, state, next |
| 85 | school, child, parents, parent, teacher, close, home, keep, open, student |
| 86 | game, play, player, sport, nrl, football, afl, season, club, golf |
| 87 | student, online, learn, school, work, home, university, teacher, teach, class |
| 88 | day, work, home, friend, one, time, family, today, child, mother |

**Table 1. Australian dataset topic word sets.** n=31**,** *k*=90.

| **Topic** | **Topic word set** |
| --- | --- |
| 2 | medium, information, news, post, please, share, read, social, tweet, report |
| 12 | test, positive, result, day, case, number, symptom, laboratory, capacity, negative |
| 14 | physical distance, distance, walk, keep, away, outside, maintain, practice, one, foot |
| 17 | women, men, group, pandemic, age, gender, child, vulnerable, affect, shelter |
| 20 | watch, movie, show, episode, tv, listen, video, podcast, time, netflix |
| 23 | day, weather, spring, water, outside, fire, snow, down, today, time |
| 28 | mask, ppe, supply, medical, worker, equipment, hospital, ventilator, help, send |
| 29 | hospital, patient, doctor, test, care, symptom, call, bed, health, medical |
| 34 | stay, home, safe, please, keep, physical distance, work, family, healthy, self isolate |
| 40 | ship, cruise ship, passenger, quarantine, japan, travel, day, america, return, princess cruise |
| 41 | call, phone, please, email, send, message, help, try, information, line |
| 42 | centre, library, school, public, city, facility, shut down, gather, theatre, gym |
| 45 | border, close, travel, citizen, country, america, open, trudeau, allow, usa |
| 49 | care, home, long term, resident, facility, death, staff, senior, ltc, outbreak |
| 50 | room, house, hotel, space, shelter, homeless, move, place, office, table |
| 51 | hand, touch, wash hand, clean, wash, cough, face, surface, water, wipe |
| 53 | mask, wear, face, protect, public, medical, glove, spread, doctor, one |
| 55 | gather, event, cancel, large, easter, group, party, church, year, celebrate |
| 59 | close, business, work, open, essential, service, employee, non essential, store, home |
| 61 | hair, wear, day, cut, red, shirt, clothes, pant, dress, black |
| 65 | inmate, release, prison, jail, criminal, court, immigration, lawyer, prisoner, hear |
| 67 | flight, travel, quarantine, self isolate, day, return, airport, screen, home, china |
| 73 | park, walk, close, city, street, drive, bus, car, physical distance, toronto |
| 78 | child, school, parent, teacher, close, learn, home, student, work, family |
| 79 | student, school, class, university, close, online, cancel, college, work, year |
| 86 | province, emergency, government, order, act, measure, fine, health, public, rule |

**Table 2. Canadian dataset topic word sets.** n=26**,** k=90.

| **Topic** | **Topic word set** |
| --- | --- |
| 1 | back, shoulder, deep, bump, stand, side, wheel, keep, nose, head |
| 6 | law, power, police, gardai, court, right, lock down, government, state, restriction |
| 9 | day, time, home, today, house, eat, back, one, drink, work |
| 10 | time, great, watch, child, video, keep, book, day, live, free |
| 14 | dog, walk, pet, day, foster, vet, five, money, life, michel |
| 15 | fuck, stop, live, lock down, one, tell, country, down, time, try |
| 16 | school, child, student, parent, close, work, teacher, home, learn, online |
| 21 | stay, home, keep, please, social distance, safe, work, help, stay safe, spread |
| 22 | game, sport, club, gaa, football, cancel, postpone, match, italy, player |
| 23 | police, call, army, women, emergency, fire, service, men, gardai, report |
| 25 | walk, social distance, home, kilometer, park, car, today, dublin, work, road |
| 26 | traffic, reduce, speed, road, limit, less, high, increase, risk, pressure |
| 28 | day, light, hope, beautiful, today, back, thank, little, walk, one |
| 29 | pray, church, mass, day, john, dublin, michael, jesus, funeral, priest |
| 33 | test, day, result, wait, positive, symptom, doctor, case, contact, hse |
| 36 | close, pub, school, cancel, open, shut, government, parade, patrick day, gather |
| 37 | week, day, last, next, time, two, work, year, ago, month |
| 40 | family, friend, love, mother, one, home, today, child, day, father |
| 41 | ppe, supply, mask, china, medical, equipment, staff, ventilator, hospital, thank |
| 43 | travel, flight, italy, airport, country, dublin, stop, return, border, allow |
| 44 | due, postpone, march, cancel, date, plan, month, july, event, year |
| 46 | home, nurse, care, homeless, vulnerable, support, resident, family, centre, direct provision |
| 48 | hand, wash hand, wear, mask, glove, touch, wash, face, clean, cough |

**Table 3. Republic of Ireland dataset topic word sets.** n =23, *k*=50.

| **Topic** | **Topic word set** |
| --- | --- |
| 4 | due, cancel, play, rugby, event, sport, pandemic, postpone, cricket, game |
| 9 | stay, home, work, keep, safe, please, thank, family, help, time |
| 10 | lock down, death, country, week, case, government, one, spread, border, early |
| 11 | maori, risk, community, health, vulnerable, age, group, racism, pandemic, high |
| 13 | walk, day, drive, car, road, park, street, today, outside, good |
| 14 | police, rule, law, government, power, lock down, act, under, order, state |
| 16 | day, time, week, one, work, child, today, home, back, feel |
| 22 | ship, cruise ship, passenger, dock, surreal, ruby princess, nurse treat, board, sydney, positive |
| 23 | travel, flight, arrive, china, self isolate, border, quarantine, ban, fly, air |
| 24 | school, auckland, close, student, south, university, wellington, child, region, council |
| 25 | phone, app, data, call, track, internet, alert, text, emergency, home |
| 28 | test, health, doctor, hospital, influenza, risk, mask, medical, symptom, patient |
| 30 | supermarket, food, shop, open, buy, day, essential, store, delivery, eat |

**Table 4. New Zealand dataset topic word sets.** n=13, *k*=30.

| **Topic** | **Topic word set** |
| --- | --- |
| 7 | league, football, season, game, club, premier, player, play, fan, sport |
| 8 | police, army, military, officer, force, deploy, personnel, patrol, troop, ambulance |
| 11 | stay, home, lock down, social distance, government, please, advice, rule, follow, work |
| 12 | nhs, staff, care, worker, ppe, front line, nurse, test, health, doctor |
| 17 | day, walk, today, one, time, home, garden, house, back, work |
| 19 | keep, stay, home, safe, please, work, help, healthy, family, love |
| 24 | hair, wear, day, clothes, cut, mask, one, face, dress, head |
| 25 | police, prison, power, court, sell, law, arrest, release, prisoner, crime |
| 27 | school, child, close, parent, home, work, teacher, open, keep, key worker |
| 30 | student, school, year, university, exam, close, cancel, teach, work, online |
| 32 | flight, travel, italy, country, quarantine, airport, china, fly, back, britain |
| 38 | help, support, please, community, local, vulnerable, volunteer, group, time, donate |
| 41 | play, game, door, behind, football, knock, player, match, season, club |
| 43 | ppe, ventilator, government, europe, supply, nhs, equipment, order, test, mask |
| 52 | work, london, train, car, travel, bus, road, close, tube, service |
| 56 | close, pub, school, restaurant, avoid, open, shut, government, down, social |
| 58 | home, work, day, self isolate, mother, week, family, tell, stay, friend |
| 60 | sing, wash hand, happy birthday, song, time, music, play, whilst, dance, twice |
| 61 | shop, food, delivery, supermarket, store, order, customer, staff, online, open |
| 68 | death, condition, vulnerable, risk, elderly, health, disease, age, issue, care |
| 76 | mask, hand, wear, face, wash hand, clean, touch, wash, glove, spread |

**Table 5. United Kingdom dataset topic word sets.** n=21, *k*=80.

| **Topic** | **Topic word set** |
| --- | --- |
| 5 | test, symptom, spread, infection, test kit, number, result, case, cdc, day |
| 10 | hospital, nurse, doctor, care, worker, medical, work, help, thank, health |
| 14 | area, county, valley, lake, town, mountain, rural, san, oregon, city |
| 18 | drive, car, door, build, back, house, driver, front, seat run |
| 20 | vote, election, win, voter, democrat, poll, biden, bernie, november, primary |
| 23 | school, close, child, student, class, online, home, teacher, work, parent |
| 25 | family, child, friend, one, mother, love, death, please, home, year old |
| 43 | measure, preventative, strict, draconian, protection, policy, tariff, implement stringent, comply |
| 45 | train, subway, bus, hour, city, station, car, clean, mta, york |
| 47 | ship, cruise ship, passenger, quarantine, navy, dock, comfort, port, captain, infection |
| 50 | close, order, home, stay, state, governor, open, shut down, business, beach |
| 59 | stay, home, please, work, keep, live, safe, social distance, help, stay safe |
| 64 | wash hand, hand, cough, touch, wash, clean, mouth, sneeze, wipe, avoid |
| 69 | china, travel, border, country, spread, close, stop, flight, canada, ban |
| 71 | cough, symptom, fever, influenza, sick, cold, doctor, breathe, feel, test |
| 86 | game, play, sport, season, player, nba, fan, cancel, win, team |
| 87 | church, service, pastor, gather, religious, christian, easter, mass, pack, hold |

**Table 6. United States dataset topic word sets.** n=17, *k*=90.

| **Dataset** | ***k*** | **All topics** | **Top 50%** | **Top 80%** |
| --- | --- | --- | --- | --- |
|  |  | *μ,* *C*_NPMI, SD_ | *μ*, *C*_NPMI, SD_ | *μ*, *C*_NPMI, SD_ |
| **Australia** | 90 | 0.029 ± 0.068 | 0.079 ± 0.034 | 0.055 ± 0.041 |
| **Canada** | 90 | 0.036 ± 0.069 | 0.085 ± 0.037 | 0.061 ± 0.045 |
| **Ireland** | 50 | 0.042 ± 0.061 | 0.086 ± 0.034 | 0.063 ± 0.041 |
| **New Zealand** | 30 | 0.062 ± 0.042 | 0.093 ± 0.038 | 0.074 ± 0.040 |
| **United Kingdom** | 80 | 0.032 ± 0.061 | 0.081 ± 0.031 | 0.055 ± 0.044 |
| **United States** | 90 | 0.024 ± 0.070 | 0.074 ± 0.038 | 0.050 ± 0.044 |

**Table 7. Mean NPMI Coherence scores of topic models for each country’s dataset.** The mean scores for mean NPMI (*μ, C*_NPMI_) coherence measure, and their standard deviations (SD), calculated for: all topics modelled (*k*), the top 50%, and the top 80% of topics ranked from highest NPMI to lowest NPMI.

| **NPI Category** | **NPI** | **Australia**  (n=31) | **Canada**  (n=26) | **Ireland**  (n=23) |
| --- | --- | --- | --- | --- |
| **Personal Protection** | Hand Hygiene | - | 1 | 1 |
|  | Face Masks | 1 | 1 | - |
|  | Occupational PPE^a^ | 1 | 1 | 1 |
|  | Surface Cleaning |  | 1 |  |
| **Social Distancing** | Personal Contact | 1 | 1 | 1 |
|  | Public Spaces | 1 | - | 1 |
| **Testing and Tracing** | Testing | 2 | 2 | 1 |
|  | Contact Tracing | 1 | - | - |
| **Gathering Restrictions** | Sporting Matches | 2 | 1 | 1 |
|  | Cultural Events | 1 | 1 | 2 |
|  | Public Places | 3 | 1 | - |
| **Lockdowns** | Public Support | 3 | - | 2 |
|  | High-risk Groups | 1 | 1 | 1 |
|  | Vulnerable groups | 1 | 3 | 2 |
|  | Self-care | 1 | 2 | 4 |
|  | Legal Enforcement | 1 | - | 2 |
| **Travel Restrictions** | Travel Bans | - | - | 1 |
|  | Travel Quarantine | 2 | 3 | - |
|  | Border Closures | 2 | 1 | - |
| **Working at Home** | Non-essential Services | 4 | 3 | 1 |
|  | School Closures | 2 | 2 | 1 |
|  | Working at Home | 1 | 1 | 1 |

**Table 8.NPI topics for Australia, Canada, and Ireland.** The total number of NPI-topics for each country is given by n.

^a^ PPE: Personal Protective Equipment.

| **NPI Category** | **NPI** | **New**  **Zealand**  (n=13) | **United Kingdom** (n=21) | **United States** (n=17) |
| --- | --- | --- | --- | --- |
| **Personal Protection** | Hand Hygiene | - | 1 | 1 |
|  | Face Masks | - | - | - |
|  | Occupational PPE^a^ | - | 2 | 2 |
|  | Surface Cleaning | - | 1 | - |
| **Social Distancing** | Personal Contact | - | 2 | - |
|  | Public Spaces | - | 1 | 1 |
| **Testing and Tracing** | Testing | 1 | - | 2 |
|  | Contact Tracing | 1 | - | - |
| **Gathering Restrictions** | Sporting Matches | 1 | 1 | 1 |
|  | Cultural Events | - | - | 1 |
|  | Public Places | - | - | 2 |
| **Lockdown** | Public Support | 2 | 1 | 2 |
|  | High-risk Groups | - | 4 | 1 |
|  | Vulnerable groups | 1 | 1 | 1 |
|  | Self-care | 2 | 1 | - |
|  | Legal Enforcement | 1 | 1 | - |
| **Travel Restrictions** | Travel Bans | 1 | 1 | - |
|  | Travel Quarantine | 1 | - | 1 |
|  | Border Closures | - | - | 1 |
| **Workplace Closures** | Non-essential Services | - | 2 | - |
|  | School Closures | 1 | 2 | 1 |
|  | Working at Home | 1 | - | - |

**Table 9.NPI topics for New Zealand, the United Kingdom, and the United States.** The total number of NPI-topics for each country is given by n.

^a^ PPE: Personal Protective Equipment.
