# Supplementary material for: Public Perceptions and Attitudes Toward COVID-19 Nonpharmaceutical Interventions Across Six Countries: A Topic Modeling Analysis of Twitter Data
Source: J Med Internet Res. 2020 Sep 3;22(9):e21419. doi: 10.2196/21419 (PMC7505256; doi:10.2196/21419)
Supplement: Multimedia Appendix 5 [file jmir_v22i9e21419_app5.docx]

**Multimedia Appendix 5. NPI topic coding schema**

| **NPI** | **Description** |
| --- | --- |
| **Personal Protective Measures** | |
| *Hand hygiene* | Discussion of hand hygiene practices may include:   - Reminders or recommendations for practicing good hand hygiene. - Instructions for handwashing. - What materials to use, e.g., alcohol-based hand sanitizer, antiseptic solutions, or washing hands using soap and water. |
| *Respiratory etiquette* | Any discussion or encouragement about respiratory etiquette such as:   - Covering the nose or mouth when sneezing or coughing - Using tissues to sneeze or cough. - Using the crook of a sleeved arm to cough or sneeze, rather than using hands - Discarding used tissues. |
| *Occupational PPE* | Initially, occupational Personal Protective Equipment (PPE) is that which is used by any health care worker, carer, or those coming into direct contact with suspected or confirmed cases in a health care setting. In later months, this was expanded, e.g. the use of face masks by public transport operators. Other discussions may be about the availability, rationing, and quality of PPE, or about who should be using PPE. |
| *Face masks or face coverings* | Face masks, respirators, and cloth coverings are treated differently between countries and may have different meanings or purposes, e.g., stopping the spread rather than preventing contraction of the virus. Discussion may be about:   - The different types of face coverings, e.g., face masks, surgical masks, or N95 respirators. - The groups recommended to wear these. - Accessibility of this PPE. - Opinions about the efficacy or necessity for face masks or coverings. - Government messaging or instruction about wearing face marks or coverings. |
| *Surface and object cleaning* | Discussion about cleaning, decontaminating, or sanitizing items includes what materials to use, effective practices, and what places should be cleaned. For example:   - Workplaces. - Homes. - Venues frequented by a confirmed case. - Phones and tablets. - Computers, keyboards, and desks. |
| *Other environmental measures* | Other environmental measures include those that do not fall into other categories of protective measures. These may include:   - Opening of windows and ventilation. - Temperature and humidity controls. - Installing UV lighting. - Cleaning foods and disinfecting packages and mail. - Limiting the sharing of food.   Workplaces may be recommended to hold work meetings outside and eating lunch at desks rather than lunch rooms. Schools may be recommended to conduct lessons outside where possible, prevent year groups mixing, and to stagger lunch breaks. |
| **Social Distancing Measures** | |
| *Social distancing* | Discussion of the requirement, enforcement, or recommendation for people to keep a certain distance apart, e.g., 2m. |
| *Personal contact* | Any comments about the need, or recommendations to avoid any touching, hugging, kissing, shaking hands, or other forms of contact. |
| *Cancellation of mass gatherings* | Restrictions of mass gatherings are those that cancel, or place a cap on the number of people attending large public gatherings such as:   - Sporting matches. - Religious services. - Festivals and cultural events. - Concerts. |
| *Restrictions on smaller gatherings* | Smaller gatherings include those such as workplace events or large meetings, social meetings, recreational sports or gym groups, and where people are gathering to attend or use a public service, e.g., farmers' markets, the beach, or playgrounds. |
| *Non-essential services closures* | Discussion about the closure or non-essential services may include:   - Debate about the definitions of what an 'essential services' is. - Querying if a certain business is a non-essential service or not. - When these services will be closed. - Opinions about the need for such closures. |
| *School and University closures* | Discussion about school and university closures or transition to online learning. The effects, logistics, and timing of this may also be discussed, as well as the necessity or efficacy of these closures and the impact of homeschooling on parents and teachers. |
| **Localization Measures** | |
| *Stay-at-home* | Stay-at-home-orders are also called lockdowns. Discussion may be about current or anticipated lockdowns or plans to lift lockdowns. Different countries (or states) have different levels, stages, or phases of lockdowns. Discussion may include:   - The types of exceptions for not staying home, e.g., Attending work or education, exercise, etc. - Encouragement of others to stay home. - Opinions about the severity of the lockdown. - Opinions about how the lockdown is being enforced. - The length of the lockdown. - The timeliness of the lockdown. |
| *Provisions for at-risk groups* | At-risk groups include the elderly or those with medical conditions, which make them especially vulnerable to the effects of the virus. Provisions for at-risk individuals may consist of encouraged or mandatory self-isolation, banning entry to aged care facilities, or other precautionary practices. |
| *Provisions for vulnerable groups* | Vulnerable groups include those that are not in a position to take the necessary precautions to protect themselves from contracting the virus. This may include the homeless, those in prison, or those who live in densely populated dwellings. |
| *Self-Isolating* | Individuals who have tested positive for COVID-19 are required to self-isolate. In some countries (or states), this means staying at home if well enough to do so Self-isolation. Others may require positive cases to undergo their time in self-isolation at other facilities. (Note: The term self-isolation is often used interchangeably with quarantine, which is incorrect.) |
| *Quarantine* | Quarantine is a period of isolation undertaken by individuals at risk of having contracted the virus but is not known to be infected. Discussion of quarantine may relate to the reasons for this, including close contacts of confirmed cases, returning travelers from overseas or interstate, and while waiting for a test result. Quarantine may be undertaken in a state facility and may be mandatory and enforceable. |
| **Testing and Contact tracing** | |
| *Contact tracing* | This includes both manual contact tracing as well as that which is conducted through the assistance of a mobile app. |
| *Testing* | Discussion about testing, including their availability. The case criteria for testing will be different between countries and has changed over time. For example, testing may be restricted to those with a fever, cough, or shortness of breath, or if the person has been in close contact with a positive case. Other countries accept a broader range of symptoms such as a blocked nose or sinus, stuffy or running nose, sore throat, or wheezing. The experience of being tested, as well as testing waiting times and laboratory ‘backlogs’ may be discussed. Asymptomatic and sentinel testing strategies have been enacted in some countries. |
| **Travel Restrictions** | |
| *Travel warnings* | Travel warnings are issued by the relevant government departments regarding the safety of specific destinations. Discussion about travel warnings may include the changing of risk indicators or the lack of such indicators for countries or regions with high infection rates. |
| *Entry and/or Exit screening* | Entry and exit screenings are conducted at ports, airports, and border checkpoints. These may include temperature checks, thermal imaging, questioning by customs officers, questionnaires, or declarations of health. |
| *International travel restrictions* | This includes any discussion of:   - Both entry and exit travel bans. - What constitutes essential travel. - Border closures (internally and externally). - Bans on travelers from specific countries. - Quarantine for return travelers. |

**Table 1. Initial coding schema for identification of NPI related topics.**

This coding schema above informed by the following source.

World Health Organization. Non-pharmaceutical public health measures for mitigating the risk and impact of epidemic and pandemic influenza: annex: report of systematic literature reviews. *World Health Organization*; 2019. ISBN: 978-92-4-151683-9

The following sources supported the construction of this coding schema:

1. Bałazy A, Toivola M, Adhikari A, Sivasubramani SK, Reponen T, Grinshpun SA. Do N95 respirators provide 95% protection level against airborne viruses, and how adequate are surgical masks?. *Am J Infect Control*. 2006;34(2):51-57. doi: 10.1016/j.ajic.2005.08.018 PMID: 16490606
2. Centers for Disease Control and Prevention. 2020 Feb. Use of Cloth Face Coverings to Help Slow the Spread of COVID-19. Coronavirus Disease 2019 (COVID-19). URL: https://web.archive.org/web/20200515021655/https://www.cdc.gov/coronavirus/2019-ncov/prevent-getting-sick/diy-cloth-face-coverings.html [accessed 15-05-2020]
3. Chan KH, Yuen KY. COVID-19 epidemic: disentangling the re-emerging controversy about medical facemasks from an epidemiological perspective [published online ahead of print, 2020 Mar 31]. *Int J Epidemiol*. 2020; dyaa044. doi:10.1093/ije/dyaa044 PMID: 32232402
4. Department of Health. 2020 Apr 28. Information on social distancing. Coronavirus disease (COVID-19). Australian Government. URL: https://www.health.gov.au/sites/default/files/documents/2020/03/coronavirus-covid-19-information-on-social-distancing.pdf [accessed 15-05-2020]
5. Feng S, Shen C, Xia N, Song W, Fan M, Cowling BJ. Rational use of face masks in the COVID-19 pandemic. *Lancet Respir Med*. 2020;8(5):434-436. doi:10.1016/S2213-2600(20)30134-X PMID: 32203710
6. Inglesby TV, Nuzzo JB, O'Toole T, Henderson DA. Disease mitigation measures in the control of pandemic influenza. *Biosecur Bioterror*. 2006;4(4):366-375. doi:10.1089/bsp.2006.4.366 PMID: 17238820
7. McLean K, Burton W. 2020 Mar 22. So you have been asked to self-isolate or quarantine. What now? *Royal Australian College of General Practitioners.* URL: Retrieved from https://www1.racgp.org.au/newsgp/gp-opinion/so-you-have-been-asked-to-self-isolate-or-quaranti [accessed 25-05-2020]
8. Scottish Government. 2020 Mar. Coronavirus (COVID-19): Public use of face coverings. URL: https://www.gov.scot/publications/coronavirus-covid-19-public-use-of-face-coverings [accessed 15-05-2020]
