# Supplementary material for: Public Perceptions and Attitudes Toward COVID-19 Nonpharmaceutical Interventions Across Six Countries: A Topic Modeling Analysis of Twitter Data
Source: J Med Internet Res. 2020 Sep 3;22(9):e21419. doi: 10.2196/21419 (PMC7505256; doi:10.2196/21419)
Supplement: Multimedia Appendix 6 [file jmir_v22i9e21419_app6.docx]

**Multimedia Appendix 6. Exemplar tweets that illustrate the public discussion of NPIs**

| **NPI Label** | **Example Tweet** |
| --- | --- |
| **Personal Protection** | |
| *Hand Hygiene* | After #CoronaVirusNewYork #News this is a friendly reminder courtesy of @supremebarsnyc 5 steps every time 1) Wet your hands with clean,running water 2)Lather between fingers & under nails with soap 3)Scrub hands for 20+ secs. 4)Rinse hands 5) Dry with clean towels or air dry [pic] (US: Topic 64) |
| *Face Masks* | I seen a woman come out of the grocery store wearing gloves, and instead of removing them and throwing them out, sis wiped them with a dry Kleenex and kept them on. #COVIDIOT #covid19Canada (Canada: Topic 51) |
| *Occupational PPE* | If asymptomatic people can spread #COVID19, why wouldn't we ALL be wearing masks? I have no idea if I'm spreading this virus right now. Shouldn't I wear a mask in public? Seems dangerous for the WHO to say masks don't help. #COVID19Canada (Canada: Topic 79) |
| *Surface Cleaning* | Forget washing your hands, people need to be washing their phones. Waste of time touching your phone, washing your hands then touching your phone again. Phone wipes, maybe? Just make sure your phone isn’t being used to infect you #coronavirus #coronavirusuk #CoronaVirusUpdate (UK: Topic 76) |
| **Social Distancing** | |
| *Personal Contact* | This might be the most Canadian solution to social distancing yet! This pair was out for their morning walk in downtown #Dartmouth holding a hockey stick between them to stay 6 feet apart! #COVIDCanada #COVID–19 #NovaScotia #Canada (Canada: Topic 14) |
| *Public Spaces* | Cuomo: Wait for a less crowded subway car MTA: We are running rush hour trains every 8 minutes DeBlasio: Avoid densely packed subway cars MTA: This train is now out of service, please wait on the platform for the next train with double the amount of people #coronavirus #NYC (USA: Topic 45) |
| **Testing and Tracing** | |
| *Testing* | Found out a friend’s roommate is sick with THE Hallmark symptoms of coronavirus! Lack of taste and smell, cough, fever. My friend has a slight cough. AND THEY CAN’T GET TESTED! #Coronavirusnyc #TestingForCovid19 #CoronavirusUSA (US: Topic 71) |
| *Contact Tracing* | Will we get sensible questions to the PM or Bloomfield on the contact tracing app? Who is building it? Centralized or decentralized data model? Open source code for independent privacy assessment? #covid19nz (NZ: Topic 25) |
| **Gathering Restrictions** | |
| *Sporting Matches* | And THIS sport gets cancelled! And THIS sport gets cancelled! And THIS sport gets cancelled! And THIS sport gets cancelled! And THIS sport gets cancelled! And THIS sport gets cancelled! #CoronavirusUSA (US: Topic 86) |
| *Cultural Events* | Beautiful Mass at the Sacred Heart Church this morning: [URL] … even with the church doors closed due to the #Coronavirusireland #Limerick #Catholic #Ireland #TLM #lent #precautions #dontpanic #pray. Be assured of our prayers! [pic] (Ireland: Topic 29) |
| *Public Places* | In #Australia voting is compulsory! You get fined if you don’t vote! Why the hell is the Qld Gov making us vote during a pandemic? This is madness! Now who’s the #COVIDIDIOT #Covid19Australia#COVID19au #Covid_19australia #IFB #CoronavirusOubreak (Australia: Topic 82) |
| **Lockdowns** | |
| *Public Support* | Stay safe out there whanau! It’s a scary time and the best thing we can follow the advice and be kind to each other! Much love! #Covid_19nz (New Zealand: Topic 9) |
| *High-risk Groups* | If you are able to offer to help within your local area in the UK, or are vulnerable and need help, please see this list of community groups offering help and support #COVID19 #COVID—19 #coronavirusuk (UK: Topic 38) |
| *Vulnerable Groups* | @NYCMayor @BilldeBlasio @NYCMayorsOffice think about the victims of the crimes committed against them by the sex offenders and domestic violence criminals. Releasing these criminals puts lives in jeopardy and people at risk. #Survivor #COVID19 #coronavirus #NYC #FridayThoughts (US: Topic 49) |
| *Self-care* | Struggling with this whole situation, financially and mentally. Mental health issues resurfacing. Not dealing with it well at all#covid19australia #MentalHealthMatters (Australia: Topic 53) |
| *Legal Enforcement* | Really sad to see our great police force, An Garda Síochána, being misused to enforce unjust laws facing constitutional challenge…well done to Gemma O’Doherty and John Waters for standing up for all our freedom today. #LockdownIreland #COVID19ireland (Ireland: Topic 6) |
| **Travel Restrictions** | |
| *Travel Bans* | The NZ Government is placing temporary entry restrictions into New Zealand on all foreign nationals travelling from or transiting through mainland China to assist with the containment of the novel coronavirus and to protect New Zealand and the Pacific Islands from the disease.✊ (New Zealand: Topic 23) |
| *Travel Quarantine* | What good does the airline screening and quarantine do if we let a ship with more passengers than an airplane just get off and go home? Especially when there were sick people on the ship!!!! #coronavirususa #coronavirus #CDC @CDCgov @GovChristie (US: Topic 47) |
| *Border Closures* | @BillBlair US Citizens must be banned from entering into Canada with the exception of Truck Drivers/Personnel involved in logistics of Trade/Commerce & Diplomats. Banning US Citizens from entering into Canada is necessary due mess down there. #Canada #JustinTrudeau #COVIDCanada (Canada: Topic 45) |
| **Workplace Closures** | |
| *Non-essential Services* | 100 limit for people gathering indoors but shopping centres remain open but beauticians will be closed but retail stores and supermarkets will be open, not food courts... but you can buy food at the food courts for take away. #auspol #australialockdown #COVID19 (Australia: Topic 30) |
| *School Closures* | Victorian Teachers? Or do we have to wait for the Department? What if my Independent School kids are home- should I take carers leave? #whatdoido #socialisolation #COVID19Aus (Topic 85: Australia) |
| *Working at Home* | The new normal: virtual meetings & catch ups with folks at @GatewayAntarct1 Virtual cake, breakout rooms and the boss, aka #CaptKirk - @AdrianMcNZ #StarTrek background, made for an entertaining meeting! #WeCanDoThis #lockdownnz #polarGeeks (New Zealand: Topic 16) |

**Table 1. Example tweets from NPI topics.** URLs, pictures and other embedded media have been omitted.
